# Early Dutasteride Monotherapy in Patients With Elevated Serum Prostate-Specific Antigen Levels Following Robot-Assisted Radical Prostatectomy

**DOI:** 10.3389/fonc.2019.00691

**Published:** 2019-08-02

**Authors:** Chin-Heng Lu, Yen-Chuan Ou, Li-Hua Huang, Wei-Chun Weng, Yu-Kang Chang, Hung-Lin Chen, Chao-Yu Hsu, Min-Che Tung

**Affiliations:** ^1^Division of Urology, Department of Surgery, Tungs' Taichung Metroharbor Hospital, Taichung, Taiwan; ^2^Department of Medical Research, Tungs' Taichung Metroharbor Hospital, Taichung, Taiwan

**Keywords:** dutasteride, prostate cancer, robotic assistance for radical prostatectomy, biochemical recurrence (BCR), prostate specific antigen

## Abstract

**Background:** To evaluate the efficacy of early dutasteride administration in patients with a detectable prostate-specific antigen (PSA) levels after robot-assisted radical prostatectomy (RARP).

**Methods:** We retrospectively analyzed RARP patients whose pathological stage is T2a to T3b without lymph node or distant metastasis from 2007 to 2017. All patients received a daily dose of 0.5 mg of dutasteride when post-RARP PSA levels were increasing but had not achieved biochemical recurrence. PSA levels were monitored every 3 months after dutasteride administration. None of the patients received radiotherapy (RT) or androgen-deprivation therapy (ADT) before taking dutasteride. All follow-ups were begun from RARP to January 2019 or to the date of RT/ADT.

**Results:** Thirty-five patients were included in this analysis. The median followed up was 53.6 months. Twenty-two patients (62.9%) showed a PSA response in which the PSA decreased more than 10% at the first follow-up after dutasteride administration. The Pathological stage > T2 (*p* = 0.012) and positive surgical margin (*p* = 0.046) were prognostic factors for a PSA response. Twenty-three out of 35 included patients (65.7%) did not require further RT/ADT. The significant risk factor was the PSA level (*p* = 0.011) at the beginning of dutasteride treatment. The cut-off value of the PSA level to avoid further RT/ADT was 0.195 ng/ml.

**Conclusions:** Early dutasteride administration showed a significant decline in the PSA levels of patients with pathology stage >T2 and positive surgical margin in our retrospective hypothesis-generating study. If dutasteride was provided before the PSA value increased to 0.195 ng/ml after RARP, it would reduce the probability of acquirement of RT/ADT.

## Introduction

The prevalence of prostate cancer is high worldwide, especially among aging men. Radical prostatectomy (RP) or radiation therapy (RT) is frequently used to treat patients diagnosed with localized prostate cancer. Robot-assisted radical prostatectomy (RARP) is commonly used to treat localized prostate cancer (1). However, approximately 25–40% of these patients experience biochemical recurrence (BCR) within 5 years (2).

According to EAU-guidelines, BCR was defined by two consecutive rising PSA values >0.2 ng/ml for following RP (3). The management method for the prevention of BCR following primary curative treatment of prostate cancer remains disputable. Methods used to prevent BCR after RP include RT at least on the prostatic bed, complete or intermittent androgen-deprivation therapy (ADT), and observation (4). Some patients with BCR hesitated to undergo RT or ADT because of significant side effects, such as radiation cystitis, proctitis, loss of libido, impotence, hot flushes, gynecomastia, breast pain, metabolic syndrome, diabetes, cardiovascular disease, and osteoporosis (5).

Adjuvant pharmacological treatment delays the progression of increasing PSA levels with minimal side effects and does not compromise the effects of subsequent ADT or RT. Thus, adjuvant pharmacological treatment could be a more suitable option for patients with BCR to mitigate the concerns of increasing PSA levels. Several studies have focused on the effects of 5-alpha reductase inhibitors (5-ARI) in prostate cancer. The nuclear expression of 5-AR1 has been reported to be higher than that of 5-AR2 in prostate cancer cells (5). Dutasteride caused dual inhibition of 5-AR1 and 5-AR2, which subsequently suppressed DHT production more completely than finasteride, which only inhibits 5-AR2 (5). A clinical trial assessing Avodart after a radical therapy for prostate cancer study (ARTS) showed that dutasteride delayed BCR in patients after RT (6). Furthermore, Shin et al. reported that early dutasteride monotherapy in men with detectable serum PSA levels after RP led to decreased serum PSA levels as long as the patients had a low PSA nadir and Gleason score ≤6 (7).

In this study, all included patients underwent RARP, which was performed by the same experienced surgeon. Dutasteride was administered when two consecutive rising post-surgical PSA levels >0.1 ng/mL but had not reached BCR. Dutasteride was not routinely prescribed after RARP. This study aimed to investigate the efficacy of early dutasteride administration in patients who underwent RARP with an increased PSA level before BCR.

## Materials and Methods

### Study Design and Patients

Patients with prostate cancer who received dutasteride treatment after RARP from March 2007 to June 2017 were retrospectively reviewed, with 35 patients being included in this analysis. Pathological stages were from T2a to T3b without lymph node or distant metastasis. All patients were monitored from the date of RARP to January 2019 or when RT or ADT was performed. All patients received a daily dose of 0.5 mg of dutasteride when post-RARP PSA levels showed two consecutive rising PSA levels >0.1 ng/mL but had not reached BCR. PSA levels were monitored every 3 months after RARP and dutasteride usage. BCR was defined as at least two consecutive measurements of increased PSA levels >0.2 ng/mL from nadir following RARP (3). No patient underwent RT or ADT before dutasteride administration.

In this study, all patients tolerated dutasteride well till BCR. There was no patient who discontinued dutasteride due to side effects. All patients could tolerate dutasteride treatment before undergoing RT or ADT.

First, all patients were divided into two groups according to whether or not the first measurement of the serum PSA level decreased after dutasteride administration. PSA response was defined as a decrease in the serum PSA level by >10% after dutasteride administration. These patients were also defined as PSA responders.

Second, all patients were divided into two groups according to whether patients started receiving ADT, RT, or further salvage treatment due to BCR. Patients who exhibited stable PSA levels after dutasteride administration and did not require further salvage treatment were categorized as the successful group. Third, predictive factors of dutasteride treatment were identified in the responders and non-responders.

Clinical data included age when RARP was performed, body mass index (BMI), preoperative PSA level, free/total PSA ratio, postoperative PSA level, date and PSA level during dutasteride administration, prostate volume, and PSA density (PSAD). The prostate volume was determined by directly measuring the size of the prostate specimen from RARP. PSA nadir and PSA doubling time post-RARP were also assessed. Gleason scores, tumor volume (percentage of cancer cells in the whole prostate), pathologic T stage, positive surgical margins (PSM), seminal vesicle (SV) invasion, total number of resected lymph nodes (LNs), organ-confined (OC) tumor, extracapsular extension (ECE), and perineural invasion (PNI) were included as pathological data.

In our institute, adjuvant radiotherapy is not routinely performed in T3N0 patients post radical prostatectomy. We would discuss treatment options with the patients, including ADT, salvage or adjuvant radiotherapy when PSA levels increased persistently after radical prostatectomy. In this study, all patients received early monotherapy dutasteride treatment first.

### Statistical Analysis

The statistical analysis was performed by using IBM SPSS ver. 20.0 (IBM Co., Armonk, NY, USA). Chi-square test, two-sample *T*-test, and receiver operating characteristic curve (ROC curve) were used in the analysis. Univariate analysis was performed in finding predictive factors from dutasteride responders. When the *p*-value was <0.05, the outcomes were considered statistically significant.

## Results

In total, 35 patients were included in this analysis and the median follow-up was 53.6 months. Basic characteristics were presented in Table 1. The mean age for receiving RARP was 62.9 years (range, 53–81 years). The pre-operation mean of PSA was 14.37 ng/ml (range, 2.39–135.8 ng/ml). Twenty-two patients (62.9%) decreased more than 10% in serum PSA levels at the first follow up after dutasteride usage. Pathological stage >T2 and PSM were statistically significant predictive factors for a PSA response (*p* = 0.012 and *p* = 0.046, respectively). We divided the pathological stage T2 in to T2R0 and T2R1 sub levels in advance. We defined pathological stage T2R1 as clinical stage T2 with PVM. Pathological stage T2R0 means clinical stage T2 without PVM. The statistical difference of the pathological stage > T2R1 is not significant between PSA responders and non-responders.

**Table 1:** Comparison of clinicopathological variables between PSA responders vs. non-responders.

		**Total** **(*n* = 35)**	**PSA responder** **(*n* = 22)**	**PSA non-responder** **(*n* = 13)**	***p-*value**
BMI (kg/m^2^)		24.17 ± 1.77^*^	23.74 ± 2.82	24.88 ± 2.76	0.252^a^
Age (year)		62.89 ± 4.83	62.18 ± 3.85	64.08 ± 629	0.275^a^
PSA (ng/ml)		14.37 ± 22.47	11.92 ± 9.7	18.53 ± 35.75	0.415^a^
Specimen volume (ml)		33.46 ± 12.88	33.91 ± 12.84	32.69 ± 13.94	0.795^a^
TRUS biopsy Gleason score^####^		6.54 ± 1.07	6.32 ± 1.00	6.92 ± 1.12	0.174^a^
TRUS biopsy Gleason group	Group 1	19	13	6	0.283^b^
	Group 2	9	7	2	
	Group 3	3	1	2	
	Group 4	1	0	1	
	Group 5	3	1	2	
PSAD (ng/ml^2^)		0.81 ± 2.29	0.51 ± 0.85	1.32 ± 3.69	0.330^a^
Tumor_volume (%)		5.42 ± 4.74	6.32 ± 5.42	3.90 ± 3.16	0.153^a^
Total_LN (*n*)		9.43 ± 4.60	9.32 ± 5.43	9.62 ± 3.20	0.859^a^
PSA_nadir (ng/ml)		0.03 ± 0.07	0.04 ± 0.09	0.02 ± 0.02	0.479^a^
PSA when dutasteride usage (ng/ml)		0.17 ± 0.11	0.19 ± 0.11	0.15 ± 0.10	0.368^a^
Duration from RARP to Dutasteride (months)		31.94 ± 26.79	31.5 ± 30.1	32.69 ± 22.61	0.902^a^
PSA_doubling_time (months)		25.73 ± 72.44	15.29 ± 18.73	42.58 ± 117.40	0.300^a^
Pathology Gleason score		7.00 ± 0.87	6.95 ± 0.79	7.08 ± 1.04	0.876^a^
Pathology Gleason group	Group 1	9	5	4	0.571^b^
	Group 2	15	11	4	
	Group 3	6	4	2	
	Group 4	1	0	1	
	Group 5	4	2	2	
Pathology stage	T2aN0M0	2	1	1	0.141^b^
	T2bN0M0	1	0	1	
	T2cN0M0	5	1	4	
	T2R1N0M0^#^	9	6	3	
	T3aN0M0	16	13	3	
	T3bN0M0	2	1	1	
Pathology > T2^##^		27	20	7	0.012^b^^*^
Pathology > T2R1^###^		18	14	4	0.060^b^
OC (organ confined)		19	10	9	0.172^b^
ECE (extracapsular extension)		17	13	4	0.105^b^
PSM (positive surgical margin)		21	16	5	0.046^b^^*^
SV (seminal vesicle)		2	1	1	0.698^b^
PNI (perineural invasion)		25	18	7	0.077^b^

Data are presented as n or mean ± standard deviation.

a Two-sample T test;

b Chi-square test; ^c^ BMI = weight (kg)/height (m)^2^.

# Pathology T2R1: clinical stage T2 but surgical margin positive.

## Pathology > T2 including: T2R1N0M0, T3aN0M0, T3bN0M0.

### Pathology > T2R1 including: T3aN0M0, T3bN0M0.

#### TRUS biopsy Gleason score: Transrectal ultrasound guided biopsy Gleason score.

* *Statistical significance: p < 0.05*.

Table 2 showed that 17 of 22 PSA responders (77.3%) kept an acceptable PSA level without BCR and eventually avoided further RT or ADT. We referred to this as a successful outcome. However, no statistically significant risk factor was found. Only the PSA nadir (*p* = 0.070) and the PSA level at the time of taking dutasteride (*p* = 0.062) showed the possible tendency for a successful outcome.

**Table 2:** Comparison of clinicopathological variables of success with failure in responders.

		**Success in PSA responder** **(*n* = 17)**	**Failure in PSA responder** **(*n* = 5)**	***p*-value**
BMI (kg/m^2^)		23.86 ± 2.92	23.35 ± 2.71	0.730^a^
Age (year)		61.88 ± 4.03	63.2 ± 3.35	0.514^a^
PSA (ng/ml)		11.99 ± 10.18	11.65 ± 8.91	0.946^a^
Specimen volume (ml)		33.29 ± 13.26	36.00 ± 12.45	0.689^a^
TRUS biopsy Gleason score^*####*^		6.12 ± 0.86	7.00 ± 1.23	0.163^a^
TRUS biopsy Gleason group	Group 1	11	2	0.058
	Group 2	6	1	
	Group 3	0	1	
	Group 4	0	0	
	Group 5	0	1	
PSAD (ng/ml^2^)		0.57 ± 0.96	0.31 ± 0.17	0.562^a^
Tumor_volume (%)		6.68 ± 5.67	5.08 ± 4.84	0.574^a^
Total_LN (*n*)		9.35 ± 3.12	9.20 ± 2.17	0.957^a^
PSA_nadir (ng/ml)		0.02 ± 0.02	0.10 ± 0.18	0.070^a^
PSA when dutasteride usage (ng/ml)		0.16 ± 0.10	0.27 ± 0.12	0.062^a^
Duration from RARP to Dutasteride (months)		33.47 ± 32.87	24.80 ± 18.67	0.583^a^
PSA_doubling_time (months)		17.58 ± 20.12	5.55 ± 5.00	0.285^a^
Pathology Gleason score		6.82 ± 0.73	7.40 ± 0.89	0.126^a^
Pathology Gleason group	Group 1	5	0	0.483^b^
	Group 2	8	3	
	Group 3	3	1	
	Group 4	0	0	
	Group 5	1	1	
Pathology stage	T2aN0M0	1	0	0.867^b^
	T2bN0M0	0	0	
	T2cN0M0	1	0	
	T2R1N0M0^#^	4	2	
	T3aN0M0	10	3	
	T3bN0M0	1	0	
Pathology >T2^##^		15	5	0.421^b^
Pathology >T2R1^###^		11	3	0.848^b^
OC (organ confined)		8	2	0.781^b^
ECE (extracapsular extension)		10	3	0.962^b^
PSM (positive surgical margin)		12	4	0.678^b^
SV (seminal vesicle)		1	0	0.579^b^
PNI (perineural invasion)		13	5	0.230^b^

Data are presented as n or mean ± standard deviation.

a Two-sample T test;

b Chi-square test; ^c^BMI = weight (kg)/height (m)^2^.

# Pathology T2R1: clinical stage T2 but surgical margin positive.

## Pathology > T2 including: T2R1N0M0, T3aN0M0, T3bN0M0.

### Pathology > T2R1 including: T3aN0M0, T3bN0M0.

#### TRUS biopsy Gleason score: Transrectal ultrasound guided biopsy Gleason score.

^*^Statistical significance: p < 0.05.

Table 3 showed 6 of 13 PSA non-responders (46.2%) presented subsequently decreased PSA levels. These patients showed a stable PSA level without BCR during the follow-up period. A low PSA nadir (*p* = 0.042) and a low PSA level at the time of taking dutasteride (*p* = 0.007) were statistically significant prognostic factors for PSA decreasing after dutasteride prescription (Table 3).

**Table 3:** Comparison of clinicopathological variables of success with failure in non-responders.

		**Success in PSA non-responder** **(*n* = 6)**	**Failure in PSA non-responder** **(*n* = 7)**	***p*-value**
BMI (kg/m^2^)		26.00 ± 3.57	23.92 ± 1.50	0.186^a^
Age (year)		65.83 ± 7.52	62.57 ± 5.13	0.374^a^
PSA (ng/ml)		32.14 ± 51.46	6.57 ± 2.34	0.218^a^
Specimen volume (ml)		37.50 ± 17.82	28.57 ± 9.00	0.267^a^
TRUS biopsy Gleason score^*####*^		7.00 ± 1.27	6.86 ± 1.07	0.939^a^
TRUS biopsy Gleason group	Group 1	3	3	0.568^b^
	Group 2	0	2	
	Group 3	1	1	
	Group 4	1	0	
	Group 5	1	1	
PSAD (ng/ml^2^)		2.56 ± 5.41	0.26 ± 0.10	0.281^a^
Tumor_volume (%)		3.03 ± 3.66	4.64 ± 2.72	0.381^a^
total_LN (n)		8.00 ± 3.16	11.00 ± 2.71	0.92^a^
PSA_nadir (ng/ml)		0.01 ± 0.00	0.03 ± 0.03	0.042^a^^*^
PSA when dutasteride usage (ng/ml)		0.08 ± 0.05	0.21 ± 0.09	0.007^a^^*^
Duration from RARP to Dutasteride (months)		38.00 ± 27.25	28.14 ± 18.73	0.457^a^
PSA_doubling_time (months)		78.01 ± 173.50	12.21 ± 11.76	0.335^a^
Pathology Gleason score		7.50 ± 1.38	6.71 ± 0.49	0.320^a^
Pathology Gleason group	Group 1	2	2	0.202^b^
	Group 2	1	3	
	Group 3	0	2	
	Group 4	1	0	
	Group 5	2	0	
Pathology stage	T2aN0M0	1	0	0.464^b^
	T2bN0M0	0	1	
	T2cN0M0	1	3	
	T2R1N0M0^#^	2	1	
	T3aN0M0	2	1	
	T3bN0M0	0	1	
Pathology >T2^##^		4	3	0.391^b^
Pathology >T2R1^###^		2	2	0.853^b^
OC (organ confirmed)		4	5	0.853^b^
ECE (extracapsular extension)		2	2	0.853^b^
PSM (positive surgical margin)		3	2	0.429^b^
SV (seminal vesicle)		0	1	0.335^b^
PNI (perineural invasion)		2	5	0.170^b^

Data are presented as n or mean ± standard deviation.

a Two-sample T test;

b Chi-square test; ^c^BMI = weight (kg)/height (m)^2^.

# Pathology T2R1: clinical stage T2 but surgical margin positive.

## Pathology > T2 including: T2R1N0M0, T3aN0M0, T3bN0M0.

### Pathology > T2R1 including: T3aN0M0, T3bN0M0.

#### TRUS biopsy Gleason score: Transrectal ultrasound guided biopsy Gleason score.

* *Statistical significance: p < 0.05*.

**Figure 1:**
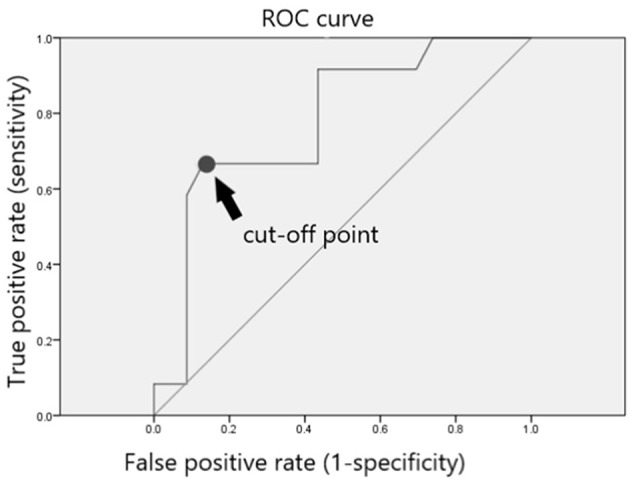
ROC curve and cut-off point for post-RARP PSA value. The ROC curve was presented in this figure. The area under the curve was 0.779 (95% confidence interval, 0.616 to 0.942, *P* < 0.007) and the cut-off value to prevent BCR was 0.195 ng/ml with the sensitivity of 66.7% and specificity of 87%. The Youden's index (sensitivity+specificity-1) was 0.537.

In total, 23 of 35 included patients (65.7%) showed a stable PSA level without BCR and did not require further RT or ADT after dutasteride treatment (Table 4). The only significant risk factor for avoiding further RT or ADT was the PSA level at the time of prescribing dutasteride (*p* = 0.011). To identify the most appropriate PSA value for the decision of dutasteride treatment, we used ROC curve to calculate the cut-off point of the PSA value. The ROC curve was presented in Figure 1. The area under the curve was 0.779 (95% confidence interval, 0.616 to 0.942, *P* < 0.007) and the cut-off value to prevent BCR was 0.195 ng/ml with the sensitivity of 66.7% and specificity of 87%. The Youden's index (sensitivity+ specificity-1) was 0.537. The result suggested that the PSA value at 0.195 ng/ml had the highest rate to prevent BCR when considering dutasteride treatment. Thus, the estimated cut-off value of the PSA to prescribe dutasteride was 0.195 ng/ml.

**Table 4:** Comparison of clinicopathological variables between success and failure.

		**Success** **(*n* = 23)**	**Failure** **(*n* = 12)**	***p*-value**
BMI (kg/m^2^)		24.42 ± 3.16	23.68 ± 2.00	0.471^a^
Age (year)		62.91 ± 5.27	62.83 ± 4.30	0.964^a^
PSA (ng/ml)		17.25 ± 27.55	8.86 ± 6.16	0.308^a^
Specimen volume (ml)		34.39 ± 14.27	31.67 ± 10.73	0.566^a^
TRUS biopsy Gleason score^*####*^		6.35 ± 1.02	6.92 ± 1.08	0.167^a^
TRUS biopsy Gleason group	Group 1	5	14	0.426^b^
	Group 2	3	6	
	Group 3	2	1	
	Group 4	0	1	
	Group 5	2	1	
PSAD (ng/ml^2^)		1.09 ± 2.85	0.28 ± 0.13	0.336^a^
Tumor_volume (%)		5.73 ± 5.40	4.83 ± 3.55	0.605^a^
total_LN (n)		9.00 ± 5.47	10.25 ± 2.56	0.461^a^
PSA_nadir (ng/ml)		0.02 ± 0.02	0.06 ± 0.12	0.085^a^
PSA when dutasteride usage (ng/ml)		0.14 ± 0.10	0.23 ± 0.10	0.011^a^^*^
Duration from RARP to Dutasteride (months)		34.65 ± 30.96	26.75 ± 17.92	0.422^a^
PSA_doubling_time (months)		33.35 ± 88.74	9.79 ± 10.09	0.390^a^
Pathology Gleason score		7.00 ± 0.95	7.00 ± 0.74	0.766^a^
Pathology Gleason group	Group 1	2	7	0.712^b^
	Group 2	6	9	
	Group 3	3	3	
	Group 4	0	1	
	Group 5	1	3	
Pathology stage	T2aN0M0	0	2	0.385^b^
	T2bN0M0	1	0	
	T2cN0M0	3	2	
	T2R1N0M0^#^	3	6	
	T3aN0M0	4	12	
	T3bN0M0	1	1	
Pathology >T2^##^		19	8	0.286^b^
Pathology >T2R1^###^		13	5	0.404^b^
OC (organ confirmed)		12	7	0.728^b^
ECE (extracapsular extension)		12	5	0.555^b^
PSM (positive surgical margin)		15	6	0.383^b^
SV (seminal vesicle)		1	1	0.630^b^
PNI (perineural invasion)		15	10	0.260^b^

Data are presented as n or mean ± standard deviation.

a Two-sample T test;

b Chi-square test; ^c^BMI = weight (kg)/height (m)^2^.

# Pathology T2R1: clinical stage T2 but surgical margin positive.

## Pathology > T2 including: T2R1N0M0, T3aN0M0, T3bN0M0.

### Pathology > T2R1 including: T3aN0M0, T3bN0M0.

#### TRUS biopsy Gleason score: Transrectal ultrasound guided biopsy Gleason score.

* *Statistical significance: p < 0.05*.

## Discussion

The major finding in this study was that 62.9% patients who were administered dutasteride showed a decreased PSA level of >10% at the first follow-up. Statistical analyses revealed that a pathological T stage of >T2 and PSM were significant predictive factors for PSA response. PSM indicates an increased risk of residual prostate cancer or benign prostate cells, which might occur due to increased PSA levels after RARP. Our results showed that dutasteride (5-ARI) could more effectively suppress PSA levels in patients with PSM. Dutasteride has been suggested to primarily prevent the production of PSA from residual prostate cancer cells.

Previous studies showed that patients with pT2 and PSM who were known as pT2R1 have a similar risk of BCR compared with those who have pT3a and negative SM (pT3aR0) (8, 9). For example, Abdullah et al. reported that patients with pT2R1 and pT3aR0 had similar risks of BCR (8). Our study showed that the pathological T stage of >T2 and no pathological T stage of >T2R1 showed a significant difference for PSA response. This study showed that patients with pT2R1 have more similar BCR risks to pT3 than patients with pT2, which was consistent with the findings of previous studies (8, 9). Moreover, our study showed that 65.7% of patients who were administered dutasteride showed stable PSA levels without BCR, indicating that they did not require further ADT or RT to control PSA levels. A significant risk factor in preventing further ADT or RT was the PSA level during dutasteride administration. The cut-off value of the PSA level was 0.195 ng/ml.

The adjuvant pharmacological treatment potentially delays the progression of PSA relapse (10). Previous clinical trials investigated the effects of finasteride, exisulind, rosiglitazone, celecoxib, dietary supplements, and phytotherapeutic interventions (5, 10). Food intake containing certain plasma carotenoids and tocopherols might be beneficial for men with PSA-defined recurrent prostate cancer. Sulforaphane, lycopene, soy isoflavones, POMx, and Pomi-T are safe and well-tolerated treatments for patients with RARP (10). The pharmacological treatment would be an exceptional therapeutic option for patients with RARP concerned about the side effects of ADT or RT.

Recently, adjuvant pharmacological treatment trials primarily focused on patients with BCR. Shin et al. investigated the effects of dutasteride on PSA progression before BCR (7).

They studied early dutasteride monotherapy in men with a detectable serum PSA level of >0.04 ng/mL after RP but before the occurrence of BCR. Their results showed that the serum PSA level decreased in men with low nadir PSA levels and Gleason score of 6 (7). Shin et al. recommended the use of routine dutasteride after RP to prevent PSA relapse. Our study showed an inconsistent result compared with that of Shin et al. because dutasteride was not routinely prescribed to patients after RARP. Dutasteride was only administered when patients with RAPP presented with an increasing PSA from nadir before BCR. The findings of our study and those of the study by Shin et al. indicate that dutasteride helps reduce serum PSA levels in patients with RARP. However, more evidence and larger samples are necessary to evaluate the therapeutic effects of routine dutasteride for the prevention of BCR in patients who underwent RP.

In our study, 12 out of 35 patients eventually met BCR and started RT before the PSA level reached 0.5 ng/ml. Several therapies for PSA relapse after RP were performed in these patients, including salvage RT (SRT) at a PSA level of <0.5 ng/ml, salvage RP, high-intensity focused ultrasound, cryosurgical ablation, or salvage brachytherapy of the prostate in radiation failures. Early SRT provides an option to reduce PSA levels. SRT has been shown to reduce PSA levels to an undetectable level in >60% of patients before reaching >0.5 ng/ml (11). ADT remains the primary choice for patients with metastatic prostate cancer.

The level of PSA nadir post radical prostatectomy is not undetectable as it is expected to reach an undetectable level 4–6 weeks post radical prostatectomy. In our study, the level of PSA nadir post radical prostatectomy is not undetectable. There were two possible reasons. Firstly, there were some PSM cases. The PSM might be due to residual prostate tissue left when nerve sparing or difficult resection of protruding medium lobe or apex especially inflammation change due to prostatitis or prostate biopsy. Secondly, micrometastasis or circulating prostate cancer cell might induce persisted detectable PSA value.

### Limitations

This study has some limitations. Because this study used a single institution database and patients were retrospectively recruited, sample biases are likely to affect our results. Another limitation is that the sexual function of these patients was not evaluated. In previous studies, some minimal adverse side effects were observed on the sexual function of patients with prostate cancer who received 5-ARIs. Sexual side effect is also an important issue for RARP treatment; thus, it cannot be ignored. Therefore, the sexual function was not analyzed in our 5-ARI study. Larger and randomized prospective studies with complete baseline and follow-up data collected by experienced RARP surgeons are required to confirm these findings.

## Conclusions

Early dutasteride monotherapy showed a significant decrease in serum PSA levels, particularly in men with pathological T stage of >T2 and PSM in this retrospective hypothesis-generating study. Moreover, if dutasteride treatment is initiated at a PSA level of <0.195 ng/ml, the requirement of further ADT or RT can be significantly avoided. Therefore, early dutasteride treatment could be considered in patients who had two consecutive rising PSA levels >0.1 ng/mL post-RARP before reaching the formal definition of BCR (i.e., PSA level of >0.2 ng/ml).

## Data Availability

The datasets for this study will not be made publicly available for ethical reason.

## Ethics Statement

This retrospective study was conducted at Tung's Taichung MetroHarbor Hospital. Certification of approval with IRB No.: 108011 (written informed consent was not required and study protocols were approved by Tung's Taichung MetroHarbor Hospital Institutional Review Board).

## Author Contributions

Y-CO and C-HL: study concepts, study design, quality control of data and algorithms. Y-CO, C-HL, L-HH, W-CW, C-YH, and M-CT: data acquisition. C-HL, Y-KC, and H-LC: data analysis and interpretation. Y-KC and H-LC: statistical analysis. C-HL and H-LC: manuscript preparation. H-LC, Y-CO, and C-HL: manuscript editing and reviewing.

### Conflict of Interest Statement

The authors declare that the research was conducted in the absence of any commercial or financial relationships that could be construed as a potential conflict of interest.
